# Superior rectus muscle insertion injury following cosmetic upper lid blepharoplasty: a case report

**DOI:** 10.1186/s12886-018-0867-2

**Published:** 2018-07-31

**Authors:** Ju-Yeun Lee, Kyuyeon Cho, Daye Diana Choi, Kyung-Ah Park, Kyung In Woo, Yoon-Duck Kim, Sei Yeul Oh

**Affiliations:** Department of Ophthalmology, Samsung Medical Center, Sungkyunkwan University School of Medicine, #81 Irwon-ro, Gangnam-gu, Seoul, 06351 Korea

**Keywords:** Superior rectus, Muscle injury, Blepharoplasty, Upper lid

## Abstract

**Background:**

Direct damage to the superior rectus (SR) muscle insertion following upper lid blepharoplasty has not been reported. We document a rare case of vertical diplopia due to direct damage to the SR muscle insertion following cosmetic upper lid blepharoplasty.

**Case presentation:**

We describe a case of 24-year-old woman with Asian eyelid. The patient had already undergone multiple cosmetic upper lid surgeries and complained of vertical diplopia immediately after her most recent surgery (levator resection with skin approach). Preoperatively, large-angle right hypotropia and severe upgaze limitation were present and noticeable ptosis was observed in the right eye. Intraoperatively, the SR muscle fibers were observed to be detached at the insertion site and severe fibrosis and adhesion surrounding the muscle was noted. After strabismus surgery, vertical strabismus was improved.

**Conclusions:**

This case can provide valuable insight to surgeons performing ptosis surgery and blepharoplasty, particularly in cases of reoperation. Surgeons should be careful while manipulating the levator muscle or resecting deep tissues not to affect the SR muscle.

## Background

Various acquired strabismus cases can result from lower lid surgery: the inferior oblique and inferior rectus (IR) muscles are likely involved [1–7]. Rarely, the superior oblique tendon can be involved in complications following upper lid blepharoplasty [4, 6, 7]. To the best of our knowledge, direct damage to the superior rectus (SR) muscle insertion following upper lid blepharoplasty has not been reported. Herein, we document a rare case of vertical diplopia due to direct damage to the SR muscle insertion following cosmetic upper lid blepharoplasty.

## Case presentation

A 24-year-old woman with Asian eyelid underwent bilateral upper lid blepharoplasty and levator tucking with skin approach for double lid formation 7 years ago. After the first surgery for cosmetic purpose, her eyelid level in the right eye was over-corrected, and thus she underwent several surgeries performed by another plastic surgeon to correct the lid level. First, she underwent removal of the levator tucking suture, but then the upper conjunctiva was prolapsed and ptosis occurred in the right eye. Prolapsed conjunctiva was resected. Subsequently, the patient underwent levator resection with skin approach for ptosis correction in the same eye. After this surgery, the patient immediately complained of vertical diplopia in the primary position that worsened in upgaze. Vertical diplopia persisted, and 2 months later, she was referred to our clinic for evaluation of strabismus.

**Fig. 1:**
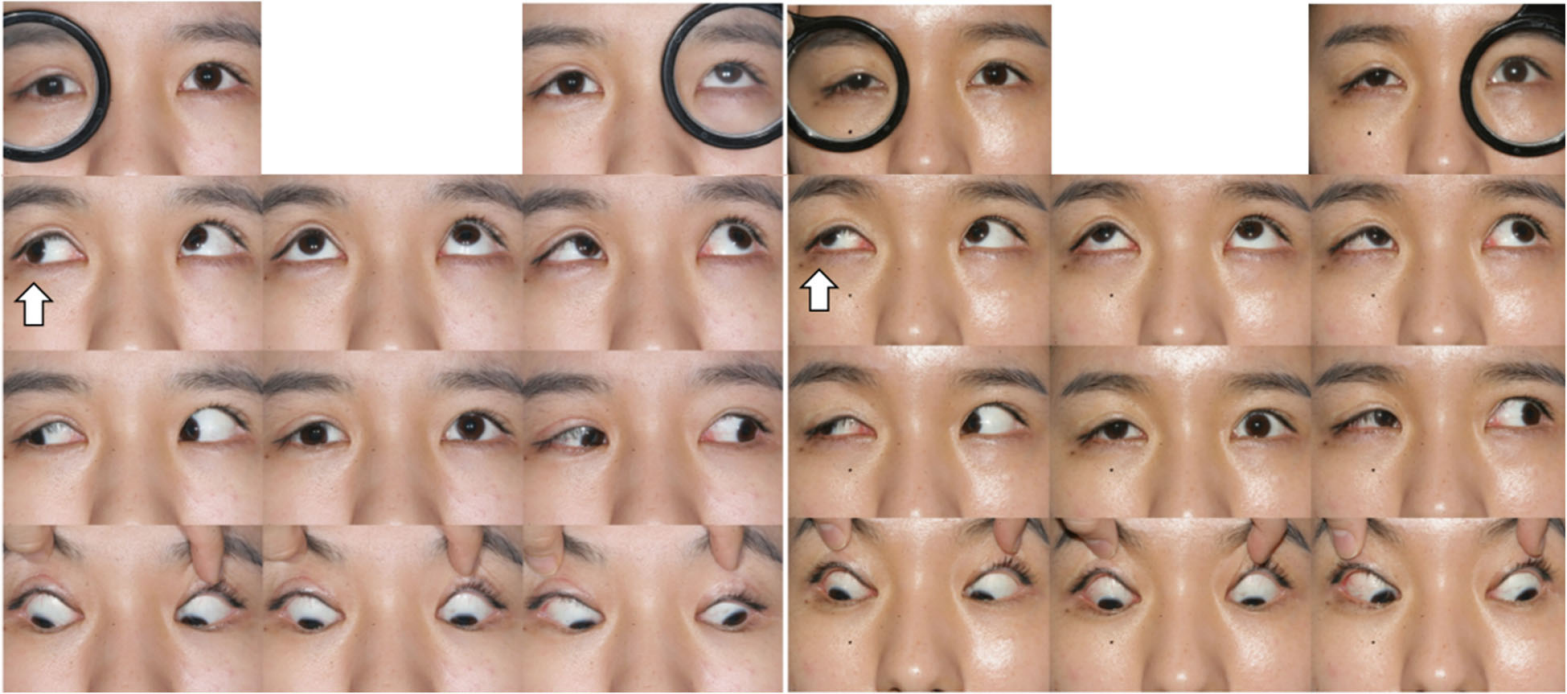
(*Left*) Preoperative 9-gaze photography. When the patient looked up, the right eye could not fully move to upward compared with the left eye. With breaking the binocular fusion (top line of the photography), noticeable vertical strabismus was observed. (arrow: right eye upgaze limitation). (*Right*) 9-gaze photography at 2 months postoperative. The upgaze limitation and vertical strabismus improved after strabismus surgery

**Fig. 2:**
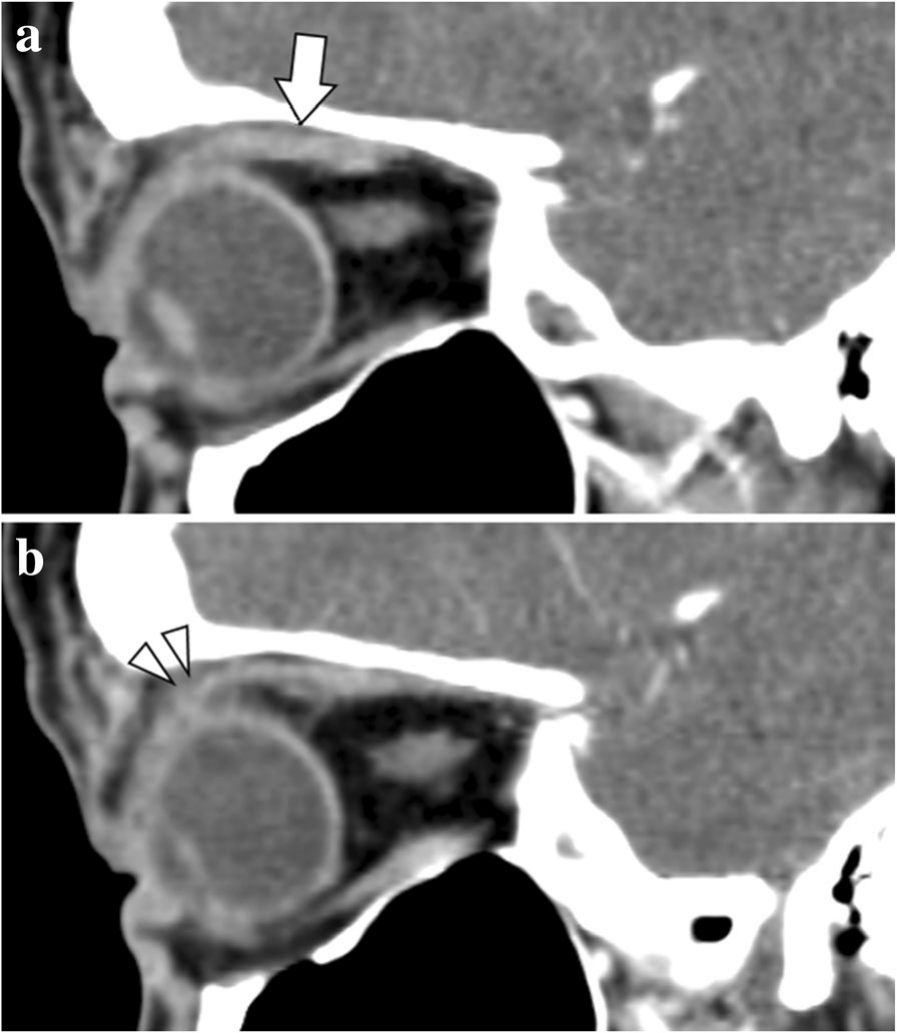
CT sagittal view scan at the first visit. **a** Suspicious enlargement of the right SR muscle was observed (arrow: the right SR muscle). **b** The infiltration around the SR muscle insertion were observed to be increased. The insertion of the SR muscle was not clearly shown in the CT scan (arrow head: ambiguous insertion of the SR muscle)

The patient underwent complete ophthalmic examination including prism and alternate cover test. We found a 25-prism-diopter (PD) right hypotropia and a 4-PD intermittent exotropia in the primary gaze, increasing to a 30-PD right hypotropia in upgaze as a consequence of the restricted upward movement of the right eye (− 2 degrees) (Fig. 1). CT scan was performed immediately, and revealed suspicious enlargement and enhancement of the right SR muscle, considered as possible damage from trauma (Fig. 2). The infiltration around the SR muscle insertion was observed to be increased, and the insertion of the SR muscle was not clearly shown in the CT. The patient was prescribed 50 mg of oral prednisolone tapered over 7 weeks. Five months later, CT was repeated; however, there was no significant change. Since there was no improvement of her hypotropia and CT scan, we elected to explore the SR muscle. Preoperatively, mild (1+) restriction in the IR muscle on the forced duction test and weakness of the SR muscle on the forced generation test were observed.

**Fig. 3:**
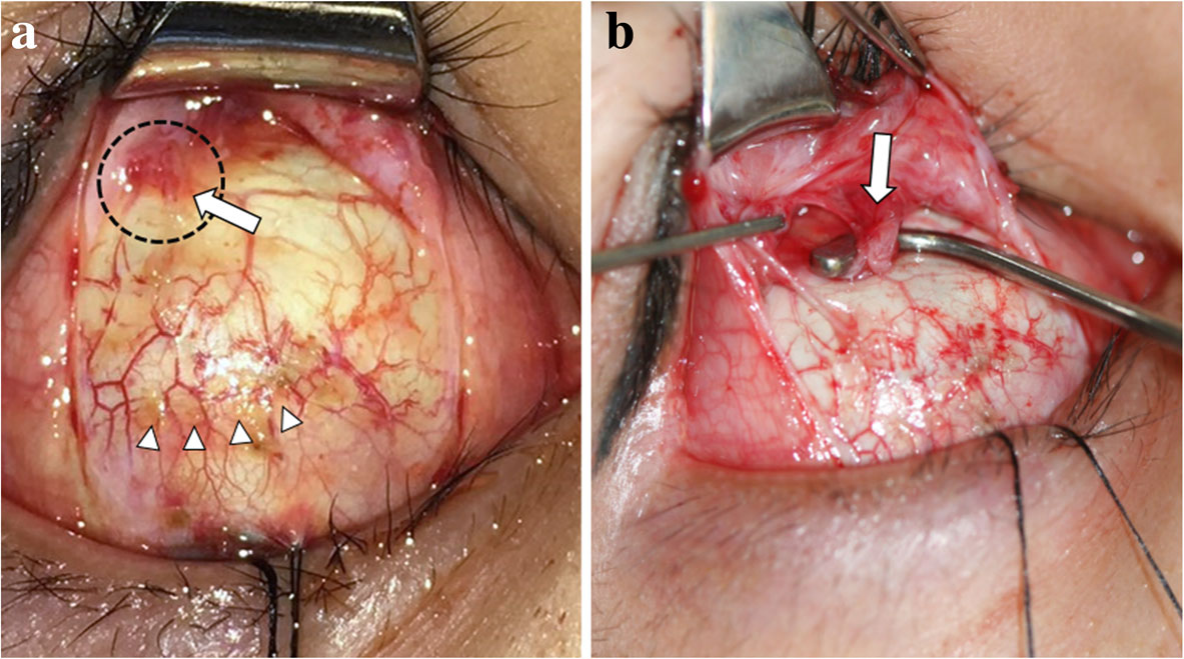
Superior rectus (SR) muscle exploration was performed through the transconjunctival approach. **a** Intraoperative findings regarding the damaged SR muscle insertion. SR muscle fibers were not observed at the original insertion site (arrow head: original insertion of the SR muscle, dotted line and arrow: remnant SR muscle fibers). **b** Instead, some SR muscle fibers were attached at the posterolateral location of the original insertion of the SR muscle (arrow: remnant SR muscle fibers)

Intraoperatively, there was neither remnant muscle fiber nor muscle capsule at the insertion site. Instead, some SR muscle fibers were attached at the posterolateral location of the original insertion of the SR muscle (Fig. 3). The superior oblique (SO) muscle was intact. There were severe adhesions around the SR muscle. Normal muscle fibers of the SR muscle were identified when tendon tissue containing radial fibers obscured the view of the muscle hook underneath it. After exploration and scar tissue removal, for nonadjustable procedures, two single-armed absorbable 6–0 polyglactin 910 sutures were passed in full thickness, double loop, locking fashion at the both margins of the remnant SR muscle fibers and perimuscular connective tissue at the anomalous attachment site. The SR muscle was sharply disinserted from the sclera and was advanced maximally to the original insertion. The muscle was sutured to the sclera at the new insertion site. Aiming to achieve initial postoperative alignment of orthotropia to 4PD right hypotropia, we additionally performed 5.5 mm IR muscle recession with a 6–0 polyglactin 910 double arm suture. No other inflammation was observed. Topical antibiotics and steroids were used postoperatively for 2 weeks.

On postoperative 1 day, a 6-PD intermittent exotropia of the primary gaze was observed and vertical diplopia was diminished. At 2 months after surgery, orthotropia was maintained in the primary gaze and there was a 2-PD right hypotropia in upgaze. Upgaze restriction of the right eye was greatly improved. There was no postoperative improvement in ptosis. At 6 months after surgery, orthotropia was observed in upgaze as well as primary gaze. At 12 months after surgery (last visit), orthotropia in all gazes was maintained.

## Discussion and conclusions

The direct damage of extraocular muscles during eyelid surgery was occasionally reported. It is demonstrated that there is a more direct route to the inferior extraocular muscle injury via transconjunctival approach than transcutaneous approach during lower lid surgery [1]. Ghabrial et al. reported that the inferior rectus and inferior oblique muscles were found to be equally injured following transconjunctival lower lid blepharoplasty [3]. Moreover, some researchers have shown that the superior oblique tendon is subject to trauma following upper lid blepharoplasty [6, 7]. Relatively, direct damage of the SR muscle is uncommon, and there was no report to describe SR muscle insertion injury during upper lid surgery.

Since the levator palpebrae superioris and the SR muscle are connected by a fibrous membrane at the medial border [8], the SR muscle can be directly damaged during resection of the levator aponeurosis. Based on the intraoperative findings in this case, strabismus was caused by direct damage to the SR muscle insertion during levator resection, likely due to aggressive excision of fat [9] or excessive resection of tissue during manipulation of the levator muscle without clear confirmation of the tissues involved. In our case, direct damage to the SR muscle insertion may have resulted from imprudent dissection of tissues that were difficult to identify due to severe adhesions related to multiple previous lid surgeries. Thus, surgeons should carefully dissect tissues under direct visualization and check eye movements during the surgery.

The SR muscle has a major role in eye elevation. Hence, damage to the SR muscle can lead to severe vertical movement disorders, and patients can suffer from intolerable diplopia in addition to cosmetic and social issues related to significant deviation of the eyeball. In this case, we obtained good results with maximal reattachment of remnant SR muscle fibers and perimuscular tissue, even though we could not locate whole muscle fibers. Whereas, ptosis was not improved with this procedure. We speculated that the remnant muscle fibers might pull the missing muscle fibers connected to the remnant muscle fibers deep into the posterior of the orbit with this advancement procedure. Based on this case, even a partial reattachment of SR muscle fibers can lead to favorable outcomes; thus it is important to find and reattach the injured muscle fibers as much as possible.

In conclusion, this case gives an important clinical significance to plastic surgeons performing upper eyelid surgery, particularly those related to the levator muscle. Surgeons should be cognizant of a risk of direct damage to the SR muscle while manipulating the levator muscle or resecting deep tissue. Using physical barriers, such as eyeball protector, can be used as a safeguard against the SR muscle injury during the surgery. In cases with ambiguous tissue anatomy due to multiple surgeries and resulting adhesions, extreme caution is needed so that damage to the SR muscle insertion can be avoided.

## Abbreviations

IR: Inferior rectus
PD: Prism diopter
SR: Superior rectus
